# C-reactive protein-to-lymphocyte ratio is a novel biomarker for predicting the long-term efficacy of ustekinumab treatment in ulcerative colitis

**DOI:** 10.1371/journal.pone.0305324

**Published:** 2024-08-29

**Authors:** Ryoji Koshiba, Kazuki Kakimoto, Noboru Mizuta, Keijiro Numa, Naohiko Kinoshita, Kei Nakazawa, Yuki Hirata, Takako Miyazaki, Kazuhide Higuchi, Shiro Nakamura, Hiroki Nishikawa

**Affiliations:** 2nd Department of Internal Medicine, Osaka Medical and Pharmaceutical University, Osaka, Japan; University of Missouri, UNITED STATES OF AMERICA

## Abstract

**Background and aim:**

Ustekinumab, a new anti-interleukin-12/23 antibody, is an effective treatment for ulcerative colitis; however, data regarding predictive factors of its efficacy are limited. Predicting treatment efficacy in advance would be useful for selecting a therapeutic agent. This study aimed to identify biomarkers that can predict the long-term outcome of ustekinumab treatment.

**Materials and methods:**

We retrospectively reviewed the records of patients with active ulcerative colitis treated with ustekinumab at Osaka Medical and Pharmaceutical University Hospital from June 2020 to January 2023. We divided patients into non-remission and remission groups, and examined whether baseline biomarkers, including C-reactive protein-to-lymphocyte ratio, and early treatment response could predict clinical remission at week 48 of ustekinumab treatment.

**Results:**

Of the 33 patients included in the study, 21 (63.6%) were in clinical remission at week 48 of ustekinumab treatment. Baseline C-reactive protein-to-lymphocyte ratio values were significantly higher in the non-remission than in the remission group. The baseline C-reactive protein-to-lymphocyte ratio value was identified as an independent prognostic factor for clinical remission at week 48 (odds ratio: 10, 95% confidence interval: 1.6–62.4, p = 0.014), with the cutoff value of 3.353 showing excellent prognostic performance (sensitivity: 71.4%, specificity: 83.3%). Furthermore, the clinical response at week 4 (odds ratio: 10, confidence interval: 1.78–56.1, p = 0.009) and that at week 8 (odds ratio: 12, confidence interval: 2.16–66.5, p = 0.005) were significantly associated with clinical remission at week 48.

**Conclusions:**

The baseline C-reactive protein-to-lymphocyte ratio value and early treatment response are useful biomarkers to predict the long-term efficacy of ustekinumab treatment.

## Introduction

Ulcerative colitis (UC) is a chronic inflammatory bowel disease (IBD) of unknown etiology characterized by erosions and ulcers in the colon. In recent years, various molecular targeted agents, including cytokine-neutralizing antibodies, have been developed for the treatment of UC. Identifying biomarkers that can predict the efficacy of each drug in advance could aid the selection of a therapeutic agent.

To date, numerous predictive biomarkers have been validated for the therapeutic efficacy of several agents used in the treatment of UC. Nishida et al. reported the usefulness of the neutrophil-to-lymphocyte ratio (NLR) as a predictor of loss of response to infliximab and clinical relapse after tacrolimus induction in UC [1, 2]. Endo et al. found that the NLR and platelet-to-lymphocyte ratio (PLR) could predict the response to systemic corticosteroid therapy in UC [3]. The C-reactive protein (CRP)-to-lymphocyte ratio (CLR) has also been reported to be a useful predictor of colectomy after infliximab treatment in acute severe UC [4]. These biomarkers are inexpensive and can be measured by routine blood tests, and can therefore be easily applied in clinical practice.

Ustekinumab (UST) is a monoclonal antibody against p40, a subunit common to interleukin (IL)-12 and IL-23. UST suppresses chronic inflammation by inhibiting IL-12, which induces T helper 1 cell response, and IL-23, which is involved in T helper 17 cell differentiation [5]. Although the UNIFI trial demonstrated the efficacy of UST induction and maintenance therapy in patients with moderate-to-severe UC [6], there are still many cases of primary non-response and loss of response to UST treatment. Hence, it would be very useful in clinical practice to predict the long-term outcome of UST treatment prior to treatment initiation. However, to the best of our knowledge, no studies have validated biomarkers for predicting the efficacy of UST treatment in patients with UC.

Therefore, the aim of this study was to identify biomarkers that can predict the long-term outcomes of UST treatment in patients with UC at an early stage.

## Materials and methods

### Study design and population

We retrospectively reviewed the records of patients with moderately to severely active UC treated with UST between March 2020 and January 2023 at Osaka Medical and Pharmaceutical University Hospital. The identification of UC was contingent upon the patient’s clinical presentation, endoscopic observations, and histological findings as well as the elimination of alternative diagnoses by the treating physician for each individual. Analyzed data included demographic and clinical characteristics, medical history, and laboratory and endoscopic findings. The partial Mayo (pMayo) scores were scored by a single gastroenterologist in an unblinded fashion based on electronic medical records.

Baseline (pretreatment) laboratory testing, including markers of systemic inflammation, was performed within one week before UST treatment initiation. Patients received a weight-based, one-time induction dose of UST of 260 mg (≤ 55kg), 390 mg (55–85 kg), or 520 mg (>85 kg) as an intravenous infusion, followed by 90-mg subcutaneous maintenance injections every 8 or 12 weeks. Response evaluation was performed at weeks 4 and 8, and every 8 weeks thereafter. All patients were followed up by physical examination and blood testing. The study was conducted in accordance with the Declaration of Helsinki, and approved by the Ethics Committee of Osaka Medical and Pharmaceutical University (protocol code 2020–112, 16 December 2020). As this was a retrospective study, the need for consent was waived by the ethics committee, with opt-out approach being apply. All data were fully anonymized before we accessed them. The data were accessed for research purposes on August 12, 2023.

### Study endpoint

The primary outcome measure of this study was clinical remission at week 48.

### Definitions

pMayo score was used to assess clinical disease activity, excluding the endoscopic subscore. The Mayo endoscopic subscore (MES) was used to assess endoscopic activity. Clinical remission was defined as a pMayo score ≤2 with each subscore ≤1. Clinical response was defined as a decrease in the pMayo score of ≥2 points relative to the baseline value, and a rectal bleeding score ≤1 or a decrease in this score of ≥1 relative to the baseline value. The remaining patients were divided in remission and non-remission groups based on whether they achieved clinical remission at week 48 of UST treatment. Individuals who discontinued UST treatment or chose alternative therapies after starting UST were classified into the non-remission group. Patients who discontinued UST treatment or chose alternative therapies after starting UST were classified into the non-remission group.

### Statistical analysis

Continuous variables were summarized as medians and interquartile ranges, and compared between the groups using the Mann–Whitney U test. Categorical variables were summarized as frequencies and percentages, and compared using the Fisher exact test. The cumulative UST treatment discontinuation rates were plotted using Kaplan–Meier plots, and differences between groups were evaluated using the log-rank test. Univariable and multivariable logistic regression analyses were employed to identify factors associated with clinical remission at week 48 of UST treatment. To evaluate the predictive performance of the identified factors, receiver operating characteristic (ROC) curves were plotted to calculate the area under the ROC curve. All statistical analyses were performed using JMP^®^, Version 15.2.1, SAS In-stitute Inc. Cary, NC, 1989–2021, USA.

## Results

### Patient characteristics

**Fig 1:**
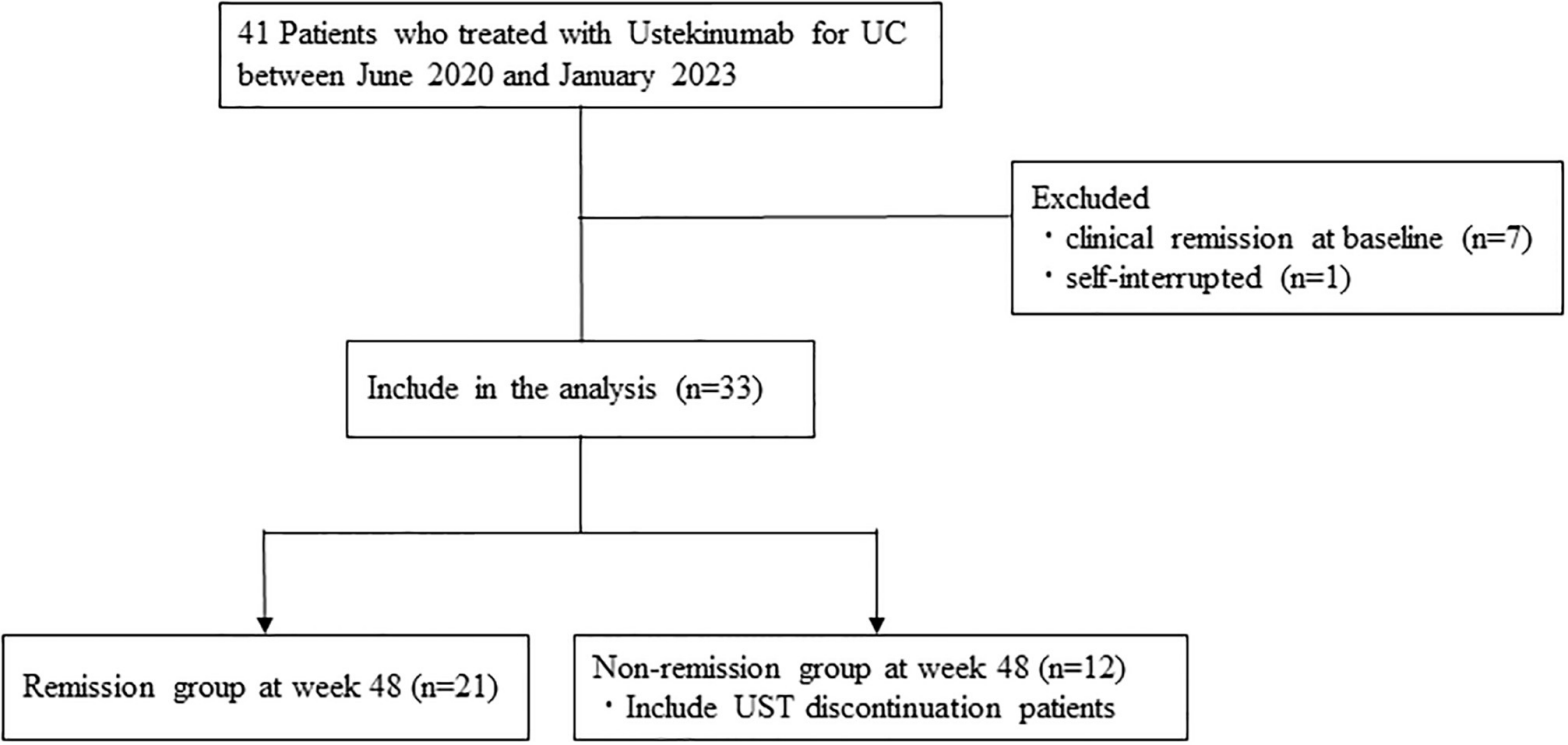
Flowchart of the study population.

A total of 33 patients (12 men and 21 women) with a median age of 36 years were included in the study. (Fig 1) All patients received UST subcutaneously every 8 weeks as maintenance therapy. The median disease duration was 86 (35–-126) months. Regarding previous treatment, 24 (72.7%) and 9 (27.2%) patients had received anti-tumor necrosis factor (TNF)-α antibody products and tofacitinib, respectively. At the start of UST treatment, 9 (27.3%) patients were taking corticosteroids and 8 (24.2%) were taking immunomodulators. The median CRP value, pMayo score, and MES were 0.43 (0.08–1.12) mg/dL, 5 (4–7), and 3 (2–3; n = 26). The baseline patient characteristics are shown in Table 1.

**Table 1:** Baseline clinical characteristics.

Number of patients, n	33
Male/Female, n	12/21
Age, year, median (IQR)	36 (30–49)
Duration of disease, month, median (IQR)	86 (35–126)
UC location; Left side/Pancolitis, n	9/24
Bio-naïve, n (%)	8 (24.2)
Prior treatment with anti-TNF, n (%)	24 (72.7)
Prior treatment with tofacitinib, n (%)	9 (27.2)
Prior treatment with vedolizumab, (%)	3 (9.1)
Concomitant 5-aminosalicylic acid, n (%)	26 (78.8)
Concomitant immunomodulator, n (%)	8 (24.2)
Concomitant corticosteroids, n (%)	9 (27.3)
Corticosteroids dependnt	23 (69.7)
Corticosteroids refractory	3 (9.1)
Partial Mayo score, median (IQR)	5 (4–7)
Mayo endoscopic Subscore (n = 26), median (IQR)	3 (2–3)
WBC, /μL, median (IQR)	7270 (5630–8910)
Hb, g/dL, median (IQR)	12.1 (10.8–13.1)
Platelet, 10^4^/μL, median (IQR)	33.3 (26.8–41.6)
CRP, mg/L, median (IQR)	0.43 (0.08–1.09)

WBC = white blood cell; CRP = C-reactive protein; IQR = interquartile range

### UST treatment efficacy up to week 48

**Fig 2:**
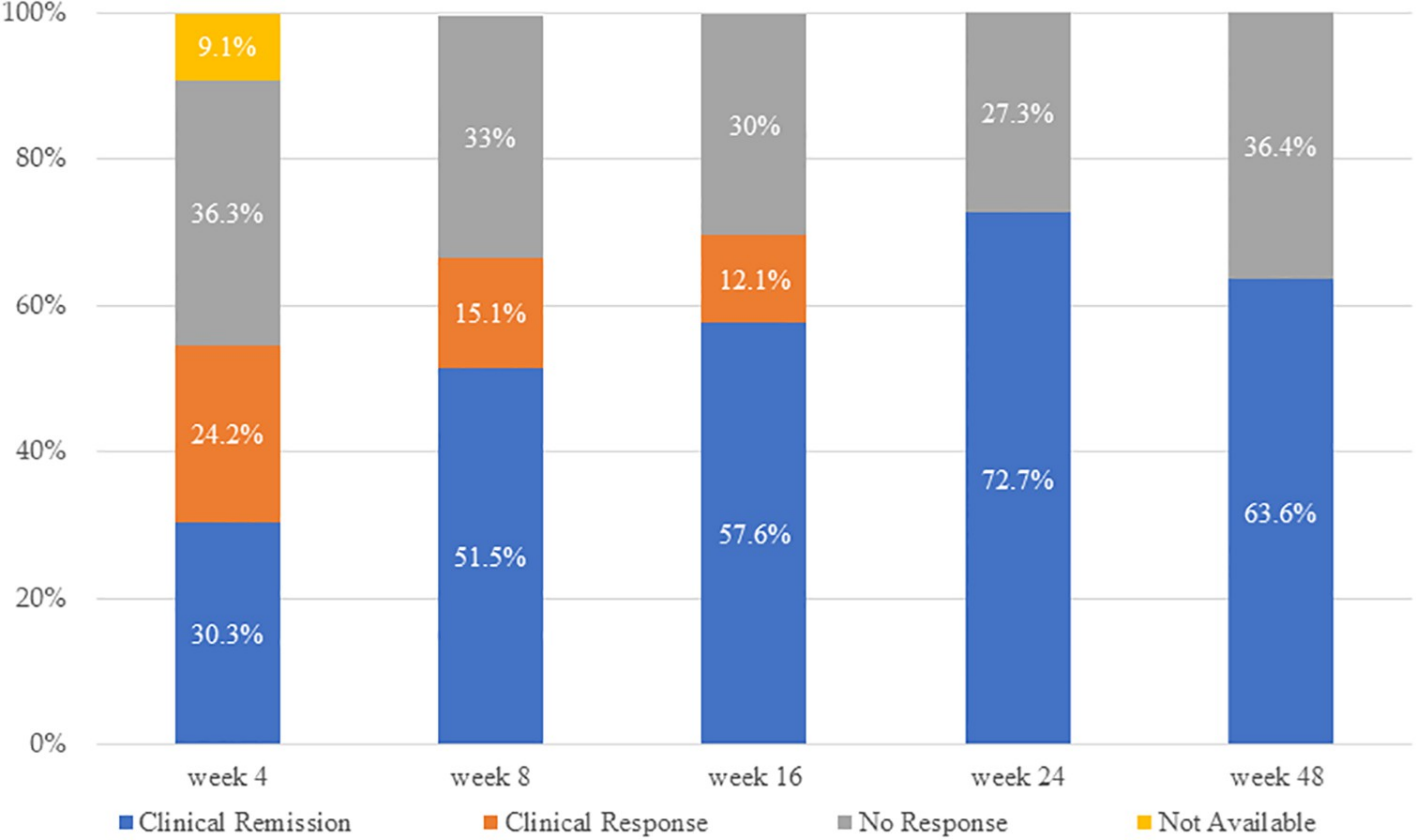
Changes in clinical remission and clinical response rates at weeks 4, 8, 16, 24, and 48.

Fig 2 demonstrates the efficacy of UST treatment up to 48 weeks. Clinical remission rates increased gradually from week 4 to week 24 (30.3%, 51.5%, 57.6%, and 72.7% at weeks 4, 8, 16, and 24, respectively), with a median clinical remission rate at week 48 of 63.6%. The clinical response rates were 54.5%, 66.7%, 69.7%, and 72.7% at weeks 4, 8, 16, and 24, respectively, and 63.6% at week 48. The UST treatment continuation rates at weeks 24 and 48 were 84.8% and 75.6%, respectively.

### Comparison between the remission and non-remission groups at week 48

Baseline clinical characteristics were compared between 21 patients in the remission group and 12 patients in the non-remission group (Table 2). Compared with those in the remission group, patients in the non-remission group had significantly shorter disease duration (p = 0.027) and significantly higher CLR values (p = 0.033). Although CRP values also tended to be higher in the non-remission than in the remission group, the difference was not statistically significant (p = 0.069). The pMayo score and MES (n = 26), as well as NLR and PLR values did not significantly differ between the two groups.

**Table 2:** Baseline characteristics between the remission group and the non-remission at week 48.

Number of patients, n (%)	Remission group 21 (63.6%)	Non-remission group 12 (36.4%)	*P*-value
Male/Female, n	6/15	6/6	0.274
Age, year, median (IQR)	36 (32–49)	41 (29–49)	0.97
Duration of disease, month, median (IQR)	111 (38–155)	56 (19–73)	0.027
UC location, Left side/Pancolitis, n	5/16	4/7	0.69
Bio-naive	7/21	1/12	0.206
History of treatment with molecular target drugs			
Anti-TNF, n (%)	14 (66.7)	10 (83.3)	0.429
Tofacitinib, n (%)	5 (23.8)	4 (33.3)	0.23
Medications for UC taken at baseline			
Aminosalicylates, n (%)	18 (85.7)	8 (66.7)	0.377
Azathioprine, n (%)	6 (28.6)	2 (16.7)	0.678
Corticosteroids, n (%)	7 (33.3)	2 (16.7)	0.429
Corticosteroids dependent, n (%)	16 (76.2)	7 (52.9)	0.433
Corticosteroids refractory, n (%)	1 (4.8)	2 (16.7)	0.538
Partial Mayo score, median (IQR)	5 (4–7)	4 (4–6)	0.746
Mayo endoscopic Subscore (n = 26), median (IQR)	2.5 (2–3)	3 (2–3)	0.302
WBC, /μL, median (IQR)	7530	7090	0.494
	(5150–9010)	(6640–8490)	
Hb, g/dL, median (IQR)	12.1	12.1	0.779
	(10.8–13.2)	(10.8–12.6)	
CRP, mg/L, median (IQR)	0.25	0.88	0.069
	(0.06–0.46)	(0.46–1.21)	
Platlet, 10^9^/L, median (IQR)	31.5	34.7	0.405
	(24.8–41.6)	(27.9–40.9)	
Neutrophil-Lymphocyte ratio, median (IQR)	3.52	4.08	0.405
	(2.18–4.56)	(3.38–5.53)	
Platelet-Lymphocyte ratio, median (IQR)	246	251	0.726
	(200–292)	(223–417)	
CRP-lymphocyte ratio, median (IQR)	1.53	7.66	0.033
	(0.53–3.96)	(4.36–11.11)	

### Predictors of clinical remission at week 48

**Fig 3:**
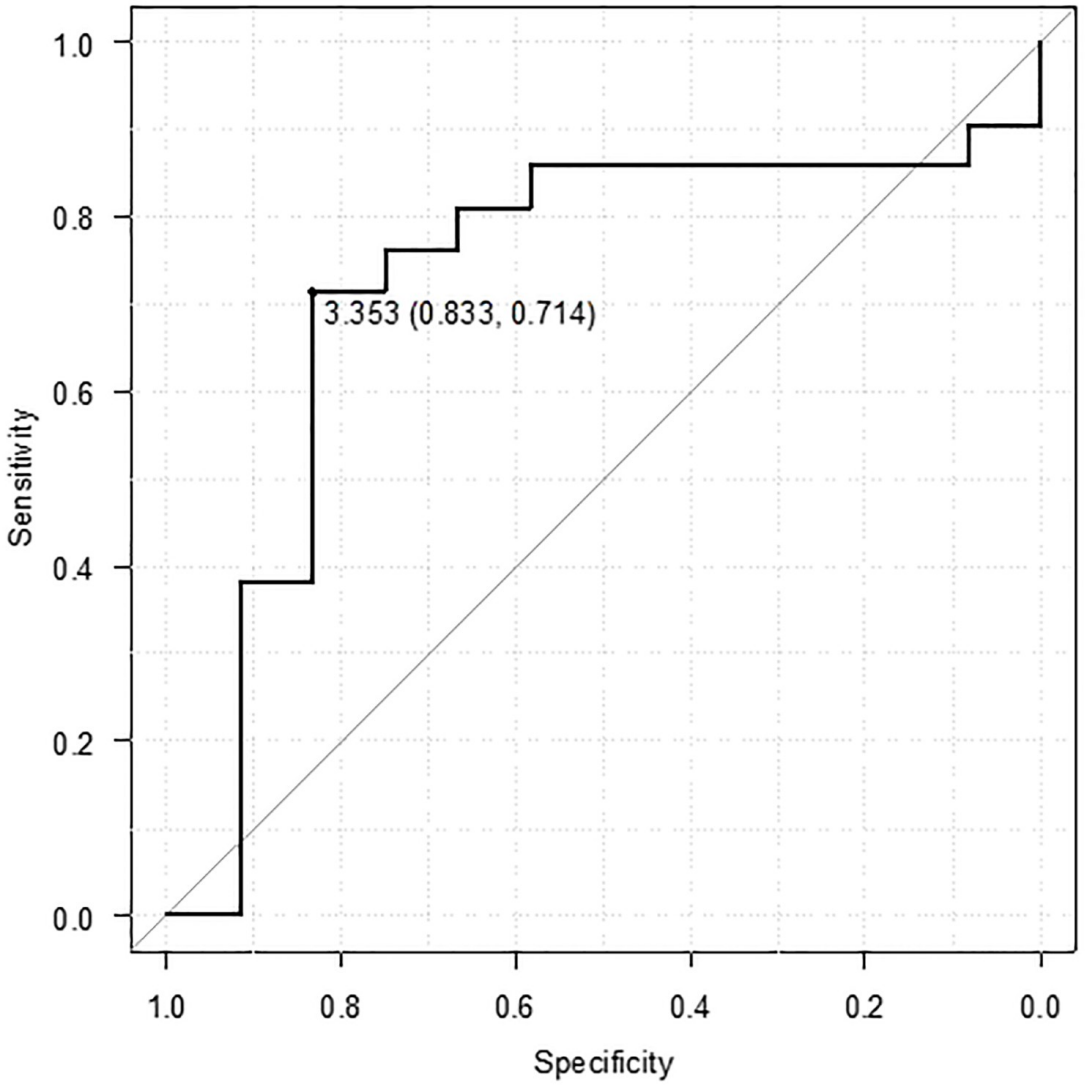
Receiver operating characteristic curve of the C-reactive protein-to-lymphocyte ratio for predicting clinical remission at week 48.

In the ROC curve analysis, the optimal threshold for the baseline CLR value associated with long-term efficacy of UST was 3.353, with an area under the curve for clinical remission at week 48 of 0.7, sensitivity of 71.4%, and specificity of 83.3% (Fig 3).

In the univariable analysis, disease duration ≥10 years (odds ratio [OR]: 6.77, 95% confidence interval [CI]: 0.73–62.8, p = 0.049) and baseline CLR value ≤3.353 (OR: 16, 95% CI: 2.59–98.8, p = 0.003) were significantly associated with clinical remission at week 48. However, in the multivariable analysis, only baseline CLR value ≤3.353 was identified as an independent predictive factor (OR: 13.5, CI: 2.09–86.7, p = 0.006).

When evaluating the UST treatment response in the early phase, the clinical response rate at week 4 (OR: 10, CI: 1.78–56.1, p = 0.009) and that at week 8 (OR: 12, CI: 2.16–66.5, p = 0.005) were significantly associated with clinical remission at week 48 (Table 3).

**Table 3:** Univariate analysis of predictive factors associated with clinical remission at week 48.

Predictive factor	Univariate analysis		Multivariate analysis	
	OR (95%CI)	*P*-value	OR (95%CI)	*P*-value
Male	0.4 (0.09–1.75)	0.22		
Duration of disease ≦ 5years	0.5 (0.12–2.13)	0.349		
Duration of disease 5-10years	0.56 (0.13–2.48)	0.445		
Duration of disease ≧ 10years	6.77 (0.73–62.8)	0.049	4 (0.35–45.7)	0.27
UC location; Pancolitis	1.6 (0.34–7.65)	0.56		
Bio-naïve	5.5 (0.59–51.6)	0.14		
History of ≧ 2 Molecular target drugs	0.86 (0.20–3.66)	0.84		
Concomitant Azathioprine	2 (0.33–12.0)	0.45		
Baseline CLR ≦ 3.353 mg/10^4^	12.5 (2.09–74.8)	0.006	10 (1.6–62.4)	0.014
Clinical response at Week 4	10 (1.78–56.1)	0.009		
Clinical remission at Week 4	9 (0.95–84.9)	0.06		
Clinical response at Week 8	12 (2.16–66.5)	0.005		
Clinical remission at Week 8	3.2 (0.73–14.4)	0.12		

CLR = CRP-lymphocyte ratio; OR = Odds ratio; CI = confidence interval

### Kaplan–Meier curve analyses

**Fig 4:**
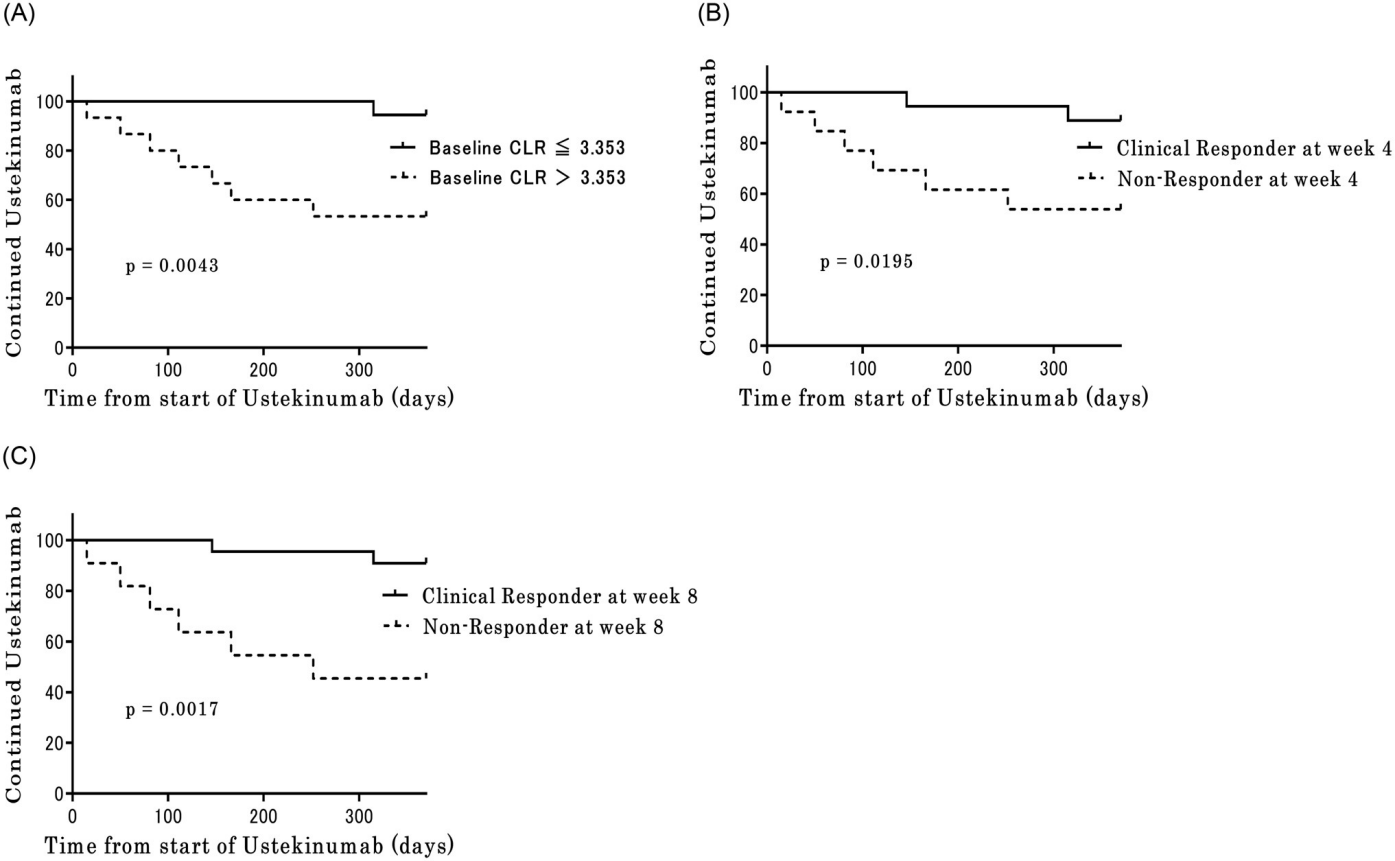
Kaplan–Meier curve analysis of the time to discontinuation of UST treatment. According to the (a) baseline C-reactive protein-to-lymphocyte ratio values, (b) clinical response rates at week 4, and (c) clinical response rates at week 8. A. Kaplan-Meier curve analysis of the time to discontinuation of UST treatment compared by Baseline CRP-lymphocyte ratio (CLR) at baseline. B. Kaplan-Meier curve analysis of the time to discontinuation of UST treatment compared by clinical response at week 4. C. Kaplan-Meier curve analysis of the time to discontinuation of UST treatment compared by clinical response at week 8.

Fig 4 depicts the survival analysis of the time to UST treatment discontinuation. Patients in the low CLR group (baseline CLR ≤3.353) showed significantly longer UST treatment duration than those in the high CLR group (baseline CLR >3.353) (p = 0.0089, Fig 4A). Furthermore, UST treatment duration was significantly longer in the clinical responder than in the non-responder group, both at week 4 (p = 0.0195, Fig 4B) and at week 8 (p = 0.0017, Fig 4C).

### UST treatment safety

Adverse events occurred in two patients (one case of skin rash and one case of upper respiratory infection requiring hospitalization); however, neither adverse event required discontinuation of UST treatment. Among nonresponders, two patients underwent elective colectomy.

## Discussion

In this study, the baseline CLR value was significantly associated with clinical remission at week 48 of UST treatment, suggesting that higher CLR values at baseline predicting lower efficacy of UST treatment can be one of the criteria for selecting therapeutic agents for UC. To the best of our knowledge, this study is the first to report pretreatment biomarkers that can be used to predict long-term UST efficacy in patients with UC. Considering the variety of therapeutic agents that have been developed for refractory UC in recent years, predicting the efficacy of a therapeutic agent prior to treatment initiation using readily available parameters would be of great clinical significance [7].

In addition to the CLR [4], other biomarkers based on the peripheral blood lymphocyte count, such as the NLR and PLR, have been reported as predictive factors for treatment efficacy in patients with UC [1–3, 8]. The lymphocyte count in the peripheral blood is decreased in active UC, which may be caused by various factors, such as infiltration of lymphocytes into the intestinal tissue, apoptosis due to autoimmune diseases, malnutrition, and leakage due to intestinal bleeding [3]. CRP, an acute-phase protein produced by hepatocytes upon stimulation with IL-6, is the most well-studied inflammatory parameter in patients with IBD [9, 10]. The CRP responses more modest in UC compared to that in Crohn’s disease, with more than 15% of patients with UC showing no CRP response [11, 12]. Chaparro et al. reported that high baseline CRP values were negatively correlated with the short-term UST response [13]. Similarly, in the current study, patients with lower long-term UST efficacy tended to have higher baseline CRP values. Although UC is a disease with a heterogeneous cytokine profile, the results suggest that UST, an anti-IL-12/23 antibody, is less effective in patients with high CRP values, who may also have high levels of IL-6. Based on these findings, the CLR, which is the ratio of CRP to the peripheral blood lymphocyte count, may be a novel biomarker that reflects the pathogenesis of UC and the mechanism of action of UST.

According to prior studies in patients with IBD, the short-term response to treatment with biologic agents, including anti-TNF antibody agents like infliximab, is predictive of the long-term efficacy [14, 15]. For UST, the short-term treatment response at weeks 8–12 has been associated with the long-term outcomes in patients with Crohn’s disease [16, 17]. In our study, the early response to UST treatment at weeks 4 and 8 was predictive of its long-term efficacy in patients with UC. Similar findings were obtained in the UNIFI study, which reported that clinical remission at week 8 of UST treatment was associated with clinical remission at maintenance week 44 [18]. Thus, the results of the present study are consistent with previously reported findings. Notably, in our study, the clinical response at week 4 was significantly associated with the long-term efficacy of UST treatment. Predicting the subsequent efficacy early after the start of UST treatment is clinically meaningful because it allows for early decision-making regarding treatment continuation.

This study has limitations due to the retrospective single-center design, which limited the sample size and may have led to selection bias. Thus, further prospective studies with a large cohort should be performed to validate the predictive value of the CLR and early treatment response for UST efficacy in patients with UC. Furthermore, as this study included only UST-treated patients with UC, it is not known whether CLR is a predictor for other advanced therapies. CLR has been reported to predict the efficacy of IFX treatment in acute severe UC and may be a predictor of different treatment modalities. Although treatment-specific biomarkers are valuable for selecting drugs, their absence may limit their utility. Further research is necessary to determine whether CLR can serve as a predictor of the effectiveness of alternative advanced therapies.

In conclusion, our study demonstrated that baseline CLR values are associated with the long-term outcomes of UST treatment in patients with UC. Therefore, this parameter should be considered when selecting a treatment agent for UC in clinical practice. In addition, early response to treatment was associated with clinical remission at week 48 of UST treatment, suggesting that a change in treatment may be considered if there is no improvement in clinical symptoms by week 8 of UST treatment.

## Supporting information

S1 Table Rates of clinical remission and clinical response at weeks 4, 8, 16, 24, and 48. (PPTX)

S2 Table Baseline characteristics between the remission group and the non-remission group at week 48. (PPTX)
