# Supplementary material for: C-reactive protein-to-lymphocyte ratio is a novel biomarker for predicting the long-term efficacy of ustekinumab treatment in ulcerative colitis
Source: PLoS One. 2024 Aug 29;19(8):e0305324. doi: 10.1371/journal.pone.0305324 (PMC11361563; doi:10.1371/journal.pone.0305324)
Supplement: S1 Table — (PPTX) [file pone.0305324.s001.pptx]

## Slide 1
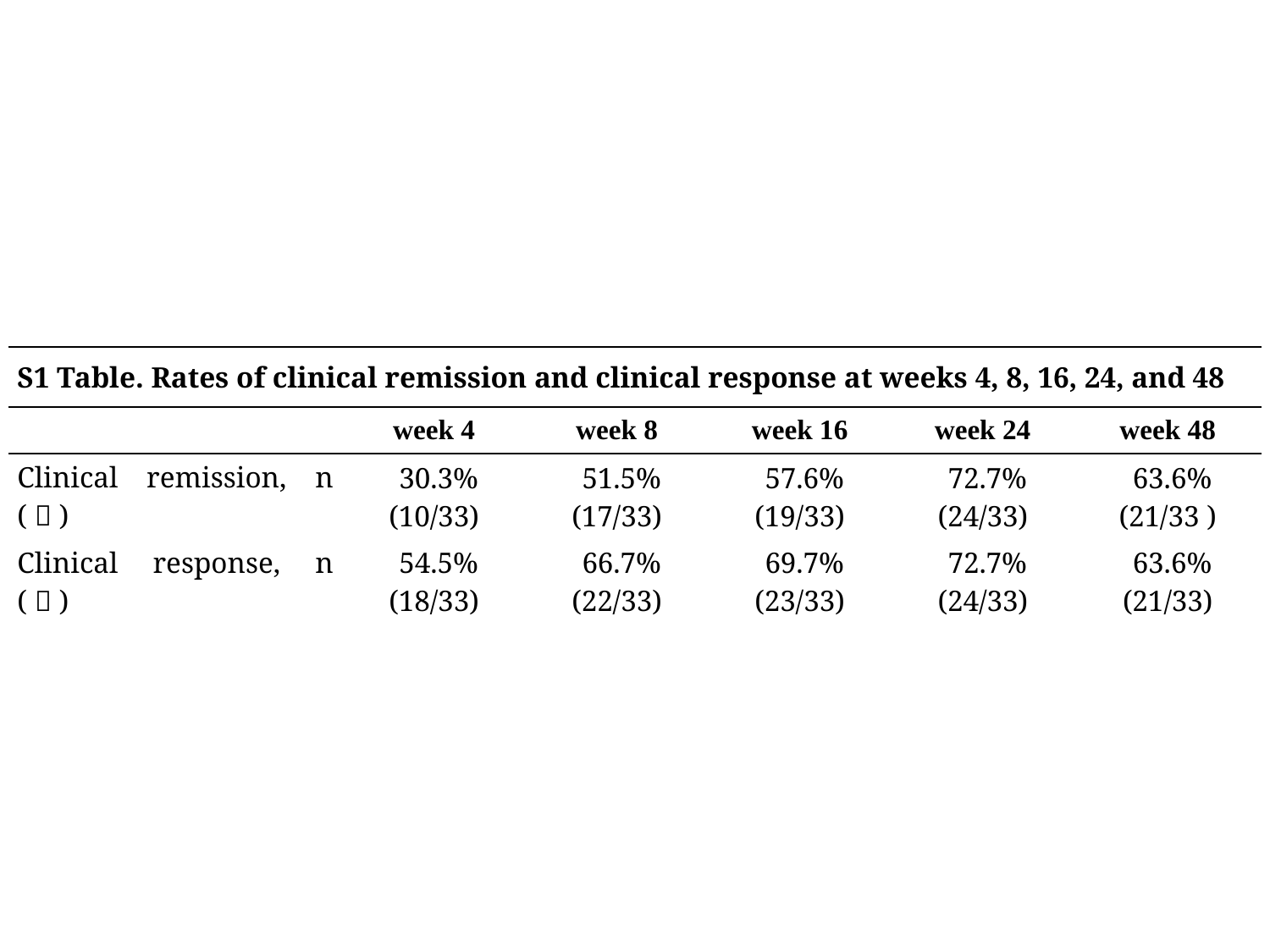

| S1 Table. Rates of clinical remission and clinical response at weeks 4, 8, 16, 24, and 48 | | | | | |
| --- | --- | --- | --- | --- | --- |
| | week 4 | week 8 | week 16 | week 24 | week 48 |
| Clinical remission, n (％) | 30.3% (10/33) | 51.5% (17/33) | 57.6% (19/33) | 72.7% (24/33) | 63.6% (21/33 ) |
| Clinical response, n (％) | 54.5% (18/33) | 66.7% (22/33) | 69.7% (23/33) | 72.7% (24/33) | 63.6% (21/33) |
