# Supplementary material for: C-reactive protein-to-lymphocyte ratio is a novel biomarker for predicting the long-term efficacy of ustekinumab treatment in ulcerative colitis
Source: PLoS One. 2024 Aug 29;19(8):e0305324. doi: 10.1371/journal.pone.0305324 (PMC11361563; doi:10.1371/journal.pone.0305324)
Supplement: S2 Table — (PPTX) [file pone.0305324.s002.pptx]

## Slide 1
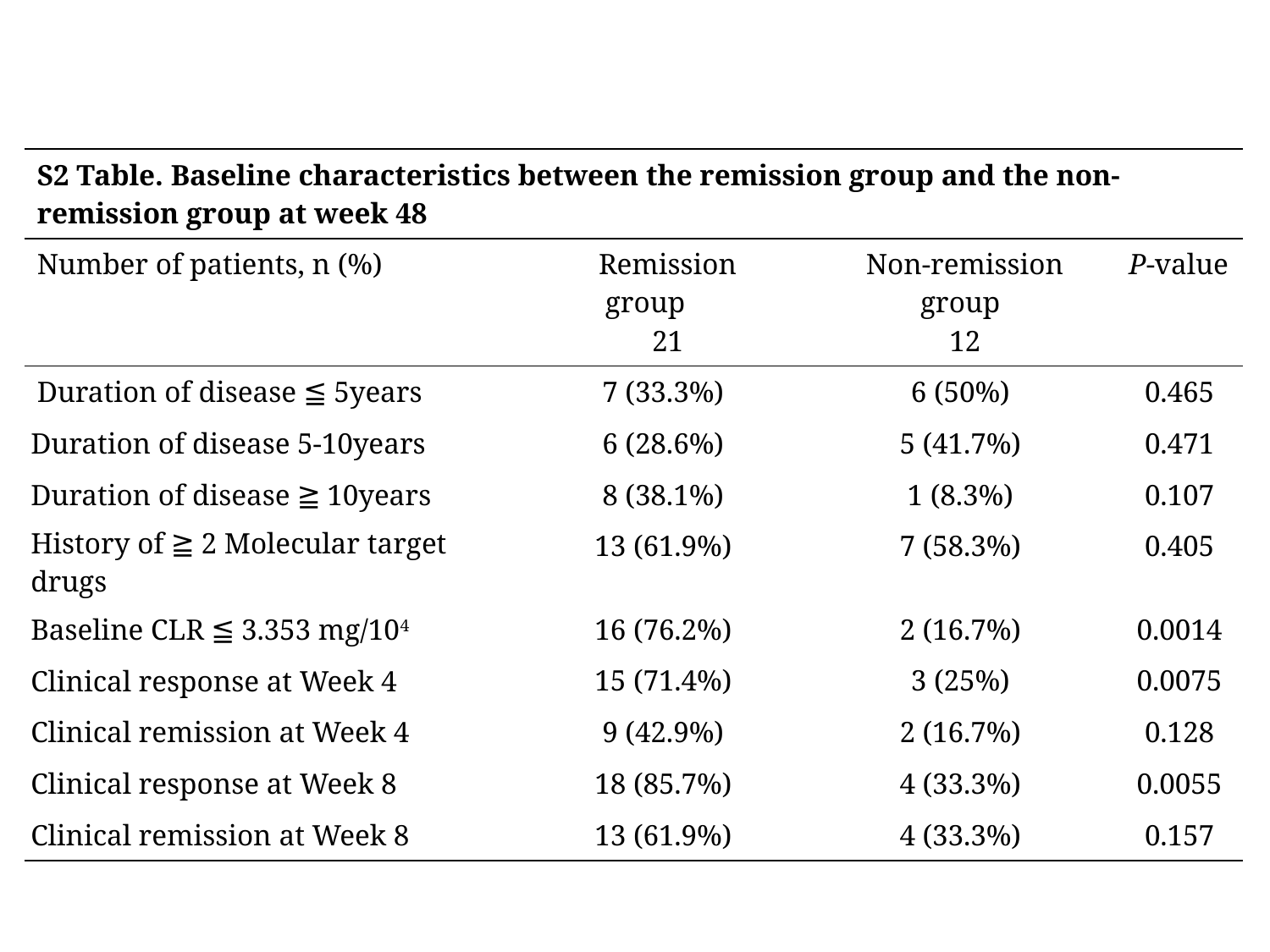

| S2 Table. Baseline characteristics between the remission group and the non-remission group at week 48 | | | |
| --- | --- | --- | --- |
| Number of patients, n (%) | Remission group　 21 | Non-remission group 12 | P-value |
| Duration of disease ≦ 5years | 7 (33.3%) | 6 (50%) | 0.465 |
| Duration of disease 5-10years | 6 (28.6%) | 5 (41.7%) | 0.471 |
| Duration of disease ≧ 10years | 8 (38.1%) | 1 (8.3%) | 0.107 |
| History of ≧ 2 Molecular target drugs | 13 (61.9%) | 7 (58.3%) | 0.405 |
| Baseline CLR ≦ 3.353 mg/104 | 16 (76.2%) | 2 (16.7%) | 0.0014 |
| Clinical response at Week 4 | 15 (71.4%) | 3 (25%) | 0.0075 |
| Clinical remission at Week 4 | 9 (42.9%) | 2 (16.7%) | 0.128 |
| Clinical response at Week 8 | 18 (85.7%) | 4 (33.3%) | 0.0055 |
| Clinical remission at Week 8 | 13 (61.9%) | 4 (33.3%) | 0.157 |
